# Development of a dual targeting scaffold of SET7/MLL inhibitor for castration-resistant prostate cancer treatment

**DOI:** 10.1016/j.gendis.2023.01.034

**Published:** 2023-03-24

**Authors:** Guodong Li, Qi Huang, Vincent Kam Wai Wong, Wanhe Wang, Chung-Hang Leung

**Affiliations:** a Institute of Chinese Medical Sciences and State Key Laboratory of Quality Research in Chinese Medicine, University of Macau, Macao SAR 999078, China; b Dr. Neher's Biophysics Laboratory for Innovative Drug Discovery, State Key Laboratory of Quality Research in Chinese Medicine, Macau University of Science and Technology, Macao SAR 999078, China; c Institute of Medical Research, Northwestern Polytechnical University, Xi'an, Shaanxi 710072, China; d Macau Centre for Research and Development in Chinese Medicine, Institute of Chinese Medical Sciences, University of Macau, Macao SAR 999078, China; e Department of Biomedical Sciences, Faculty of Health Sciences, University of Macau, Macao SAR 999078, China; f Zhuhai UM Science and Technology Research Institute, Zhuhai, Guangdong 519000, China

Histone methyltransferase enzyme SET7 and mixed-lineage leukemia protein (MLL) complex are crucial co-activators of androgen receptor (AR) and have recently emerged as potential therapeutic targets for advanced castration-resistant prostate cancer (CRPC). In this study, we described the identification of a rhodium-based hybrid complex (SM_1) as a potent blocker of AR activity via simultaneously inhibiting SET7 and MLL complex activity, which makes it a potential lead scaffold for CPRC drug development.

Two hybrid complexes **SM_1** and **SM_2** as dual inhibitors against SET7 and MLL complex were designed by coupling the Rh(III) (**SM_1**) or Ir(III) (**SM_2**) core with two 1-phenylisoquinoline CˆN ligands and one 5,6-dmphen (where 5,6-dmphen = 5,6-dimethyl-1,10-phenanthroline) NˆN ligand, which have been previously reported as SET7/9^1^ and menin modulators,^2^ respectively (Fig. 1A). The potential menin–MLL inhibition activity of complexes was evaluated using the biomolecular fluorescence complementation (BiFC) assay to study their effects on the menin–MLL interaction. To visualize the menin–MLL complex *in cellulo*, HEK293T cells were co-transfected with MLL-VC210 and VN210-menin.^3^ The hybrid Rh(III) complex **SM_1** showed higher potency than the Ir(III) complex **SM_2** towards blocking the menin–MLL interaction (Fig. S1A–C). Moreover, the binding of a small molecule fluorescent probe to the SAM-binding pocket in SET7 was tested using a fluorescence polarization (FP) assay. Encouragingly, the hybrid Rh(III) complex SM_1 strongly inhibited the SAM/SET7 interaction, with a similar potency to the positive control sinefungin, a previously reported inhibitor of SET7 (Fig. S1A, D, E). Complex **SM_1** exhibited a dose-dependent inhibition of SET7 binding with an IC_50_ *ca.* 15.5 μM in the FP assay (Fig. S1F).

**Figure 1:**
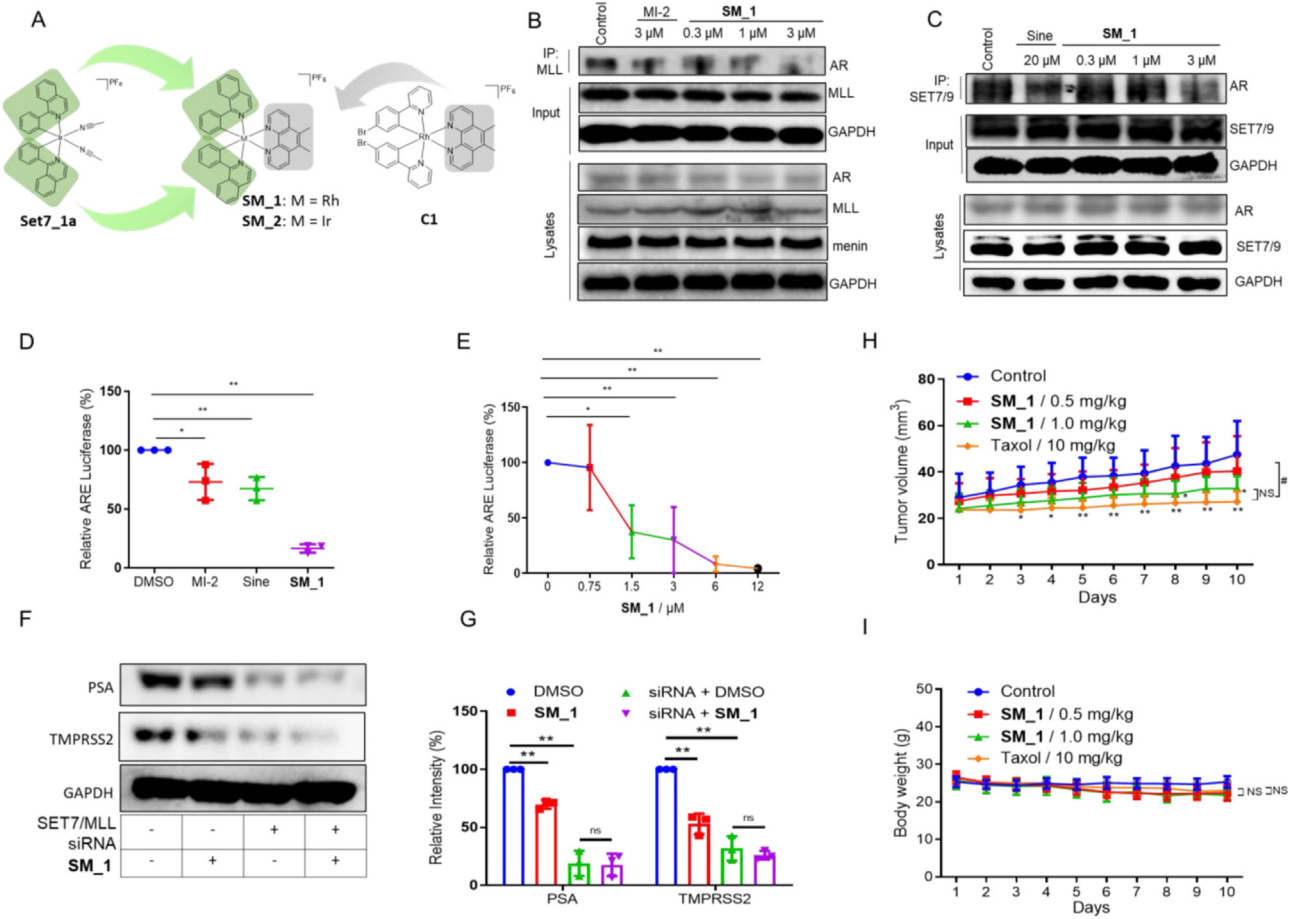
Complex **SM_1** as a dual SET7/MLL small molecule inhibitor impairs CRPC tumor growth *in cellulo* and *in vivo*. **(A)** Chemical structures of complexes **SM_1**, **SM_2**, **Set7_1a**, and **C1**. Interactions between **(B)** MLL and AR and **(C)** SET7 and AR in LNCaP cells were examined by co-IP. LNCaP cells were treated with **SM_1**, **MI-2** (3.0 μM), or sinefungin (20 μM) for 8 h. **(D)** Effect of **SM_1** (3.0 μM), MI-2 (3.0 μM), sinefungin (20 μM) on the ARE-related transcription activity in LNCaP cells by luciferase assay. **(E)** Dose–response effect of complex **SM_1** on the ARE-related transcription activity in LNCaP cells by luciferase assay. ∗*P* < 0.05, ∗∗*P* < 0.01 *vs*. DMSO group. **(F, G)** The relative amount of PSA and TMPRSS2 before or after SET7/MLL siRNA stimulation. ∗*P* < 0.05, ∗∗*P* < 0.01 SM_1 *vs*. DMSO. **(H)** Effect of complex **SM_1** on LNCaP-AR xenograft growth in castrated mice given daily with vehicle (*n* = 8), 0.5 and 1.0 mg/kg of **SM_1**, and taxol 10 mg/kg. **(I)** The body weight of castration-resistant LNCaP xenograft mice after **SM_1** treatment. ∗*P* < 0.05, ∗∗*P* < 0.01 *vs*. Control; ^#^*P* < 0.05 *vs*. Taxol. NS means not significant.

To explore whether complex **SM_1** could directly engage SET7 and MLL complex *in cellulo*, cellular thermal shift assay (CETSA) was performed in murine CRPC cell line RM-1. Menin and SET7 in cell lysates treated with 10 μM of complex **SM_1** were significantly stabilized compared with DMSO-treated controls (ΔTm of menin treated by **SM_1**: 2.0 °C; ΔTm of SET7 treated by **SM_1**: 3.0 °C) in **RM-1** cell lysates. However, there was no effect of **SM_1** on the stability of MLL and the negative control GAPDH (Fig. S2A, B). The ability of complex **SM_1** to engage menin and SET7 was also verified using a fluorescence-based protein thermal shift assay (FTS), which showed a clear shift of the melting temperature (*ca.* 3.0 °C) of purified menin and SET7 in the presence of complex **SM_1**, but not MLL (Fig. S2C). These results suggested that **SM_1** can engage menin and SET7 even in the complicated environment of cell lysates.

As a steroid hormone receptor, AR induced by its ligand 5α-dihydrotestosterone (DHT) directs the expression of target genes by binding to the androgen response elements (AREs) in PSA promoter or enhancer elements in PSA and TMPRSS2 promoters (Fig. S3A). Co-immunoprecipitation (co-IP) experiment was performed to investigate the mechanism of action of complex **SM_1** on AR activity. Incubating DHT-induced LNCaP cells with complex **SM_1** for 6 h resulted in a reduction of the amount of AR co-precipitating with MLL (Fig. 1B) or SET7 (Fig. 1C). To further evaluate the effect of **SM_1** towards the transcription of genes induced by AR, a dual luciferase reporter assay was performed. Complex **SM_1** significantly decreased the AR-directed luciferase intensity by nearly 80% at 3 μM, compared to only 27% and 34% reduction of luciferase intensity for 3 μM of MI-2 and 20 μM of sinefungin respectively (Fig. 1D). The IC_50_ value for complex **SM_1** in the luciferase assay was about 1.0 μM (Fig. 1E), which is 10 times lower than the value of the reported AR inhibitor MDV3100 (Fig. S3B). We hypothesize that complex **SM_1** reduces AR-directed transcriptional activity by inhibiting the interactions of MLL and SET7 with AR in the treated cells.

The levels of MLL and SET7 in LNCaP cells were reduced significantly after siRNA treatment (Fig. S4A, B). In the luciferase assay, **SM_1** was less able to further decrease ARE-related transcription activity in MLL/SET7 double knockdown cells compared with control cells (Fig. S4C). This suggests that the inhibition of ARE-related transcription activity induced by complex **SM_1** requires the presence of MLL and SET7 in DHT-stimulated LNCaP cells. Meanwhile, the protein levels of PSA and TMPRSS2, which are also under the control of AR, were also measured after siRNA or **SM_1** treatment (Fig. 1F, G). Similarly, complex **SM_1** showed a greater ability to reduce the levels of PSA and TMPRSS2 in control cells compared to MLL/SET7 knockdown cells.

The cytotoxicity of **SM_1** was determined in various prostate cancer cell lines (22RV1, DU145, LNCaP, and PC3) and normal cell lines (LO2 and HEK293T) using the MTT assay. **SM_1** demonstrated potent anti-proliferative effects versus the prostate cancer cell lines, particularly the CRPC cell lines 22RV1 (IC_50_ = 0.63 μM) and LNCaP (IC_50_ = 0.74 μM), while it showed lower cytotoxicity against the non-CRPC prostate cancer cell lines DU145 (IC_50_ = 1.83 μM) and PC3 (IC_50_ = 3.92 μM) and the normal human cell line LO2 (IC_50_ = 4.30 μM) (Fig. S5). Notably, the antiproliferative activity of **SM_1** towards LNCaP cells was greater than that of the reported AR inhibitor MDV3100 (IC_50_ = 19.05 μM). We suspect that the cytotoxicity displayed by **SM_1** could be associated, at least in part, with the disruption of the interaction between MLL/SET7 and AR *in cellulo*.

To evaluate the nature of cell death induced by **SM_1**, the TUNEL assay was performed. **SM_1** (3 μM) induced more apoptosis compared with MDV3100 (10 μM) or vehicle groups in both LNCaP and 22RV1 cells (Fig. S6). Furthermore, flow cytometry results indicated that both **SM_1** and MDV3100 increased cells in the G2/M phase and lowered cell counts in the G0/G1 phase in a dose-dependent fashion, indicating that these compounds induce G2/M arrest (Fig. S7), which is consistent with the G2/M arrest effect of MDV3100 and AR inhibitor in previous reports.^4^

The effect of **SM_1** on prostate cancer growth was evaluated in a mouse xenograft model of CRPC. Paclitaxel (Taxol), a clinical chemotherapeutic drug for CRPC,^5^ was used as a positive control. Compared to the control group, daily intraperitoneal (IP) injection of complex **SM_1** (1.0 mg/kg) led to inhibition of LNCaP tumor volume showing the same effect as positive drugs although 0.5 mg/kg of complex **SM_1** did not show the same effect (Fig. 1H). The reduction of tumor volume by **SM_1** was not significantly different from paclitaxel (10 mg/kg) over the course of treatment. Besides, there was also no significant difference in weight loss between the **SM_1** and paclitaxel treatment groups (Fig. 1I). These results demonstrate that complex **SM_1** exhibits comparable anti-tumor activity as compared with paclitaxel for CRPC treatment.

Collectively, our study demonstrated the potential of the SET7/MLL dual-targeting inhibitor **SM_1** for CRPC treatment through AR activity inhibition. To our knowledge, **SM_1** represents the first metal-based dual inhibitor of MLL/SET7, and we anticipate that the hybrid complex **SM_1** will serve as a potential scaffold for the development of CRPC therapeutic agents.

## Conflict of interests

The authors declare no conflict of interests.

## References

[1] Li G., Li D., Wu C. (2022). Homocysteine-targeting compounds as a new treatment strategy for diabetic wounds via inhibition of the histone methyltransferase SET7/9. Exp Mol Med.

[2] Zhong H.J., Wang W., Zhou W. (2023). Development of an orally bioavailable selective inhibitor of the menin-MLL. Genes Dis.

[3] Bellón-Echeverría I., Carralot J.P., del Rosario A.A. (2018). MultiBacMam Bimolecular Fluorescence Complementation (BiFC) tool-kit identifies new small-molecule inhibitors of the CDK5-p25 protein-protein interaction (PPI). Sci Rep.

[4] Pilling A., Kim S.H., Hwang C. (2022). Androgen receptor negatively regulates mitotic checkpoint signaling to induce docetaxel resistance in castration-resistant prostate cancer. Prostate.

[5] Gan L., Chen S., Wang Y. (2009). Inhibition of the androgen receptor as a novel mechanism of taxol chemotherapy in prostate cancer. Cancer Res.

