# Supplementary material for: Development of a dual targeting scaffold of SET7/MLL inhibitor for castration-resistant prostate cancer treatment
Source: Genes Dis. 2023 Mar 24;10(6):2260–2. doi: 10.1016/j.gendis.2023.01.034 (PMC10404973; doi:10.1016/j.gendis.2023.01.034)
Supplement: Multimedia component 1 [file mmc1.pdf]

Supplementary Materials

Development of a dual targeting scaffold of SET7/MLL inhibitor for castration-resistant prostate cancer treatment

Guodong Li,[a,f,1] Qi Huang,[b,1] Vincent Kam Wai Wong,*[b] Wanhe Wang,*[c] Chung-Hang Leung*[a,d,e,f]

[a] *Institute of Chinese Medical Sciences and State Key Laboratory of Quality Research in Chinese Medicine, University of Macau, Macao SAR, 999078, China*

[b] *Dr. Neher's Biophysics Laboratory for Innovative Drug Discovery, State Key Laboratory of Quality Research in Chinese Medicine, Macau University of Science and Technology, Macao SAR 999078, China*

[c] *Institute of Medical Research, Northwestern Polytechnical University, Xi'an, Shaanxi 710072, China*

[d] *Macau Centre for Research and Development in Chinese Medicine, Institute of Chinese Medical Sciences, University of Macau, Macao SAR 999078, China*

[e] *Department of Biomedical Sciences, Faculty of Health Sciences, University of Macau, Macao SAR 999078, China*

[f] *Zhuhai UM Science and Technology Research Institute, Zhuhai, 519000 (China)*

[1] *These authors contributed equally to this work.*

* Corresponding authors. Vincent Kam Wai Wong (bowaiwong@gmail.com), Wanhe Wang (whwang0206@nwpu.edu.cn), Chung-Hang Leung (duncanleung@um.edu.mo).

Supplementary Methods

Chemicals and reagents

Antibodies was purchased from Cell Singnaling Technology. Luciferase reporter assay system was purchased from Promega. Compound MDV3100 was purchased from Beyotime. All other reagents and chemicals were obtained commercially and used exactly as received.

General experimental.

Compound MDV3100 was purchased from Beyotime (Shanghai, China). Fetal bovine serum (FBS) and Dulbecco's Modified Eagle's Medium (DMEM) were available from Gibco BRL. Mass spectrometry was performed at the Mass Spectroscopy Unit at the Department of Chemistry, Hong Kong Baptist University, Hong Kong (China). Deuterated solvents for NMR purposes were obtained from Armar and used as received.^{1}H and ^{13}C NMR were recorded on a Bruker Advance 400 spectrometer operating at 400 MHz (^{1}H) and 100 MHz (^{13}C). ^{1}H and ^{13}C chemical shifts were referenced internally to solvent shift (Acetonitrile-d_3: ^{1}H, δ1.94, ^{13}Cδ118.7; Acetone-d_6: ^{1}H δ2.05, ^{13}Cδ29.7). Chemical shifts (d) are quoted in ppm, the downfield direction being defined as positive. Uncertainties in chemical shifts are typically ±0.01 ppm for ^{1}H and ±0.05 for ^{13}C. Coupling constants are typically ± 0.1 Hz for ^{1}H-^{1}H and ±0.5 Hz for ^{1}H-^{13}C couplings. The following abbreviations are used for convenience in reporting the multiplicity of NMR resonances: s, singlet; d, doublet; t, triplet; q, quartet; m, multiplet. All NMR data was acquired and processed using standard Bruker software (Topspin).

General preparation of the precursor complexes $M_2(C$^$N)_4Cl_2$

$MCl_3 \cdot xH_2O$ (where M = Rh or Ir) was heated to 130 °C with 2.1 equivalents of the cyclometallated ligands in the mixture of methoxyethanol:H_2O (v/v, 3:1) under a nitrogen atmosphere overnight. The reaction was cooled to room temperature, and the product was filtered and washed with three portions of deionized water and then three portions of ether to yield the corresponding cyclometalated dichloro-bridged dimers (M[C^N]$_2$Cl$_2$) for next step.

General synthesis of M(C^N)$_2$(N^N)PF$_6$

The complexes were then synthesized using the prepared M$_2$(C^N)$_4$Cl$_2$ and the corresponding N^N ligand. A suspension of M[C^N]$_2$Cl$_2$ (0.06 mmol) and the N^N ligand (0.12 mmol) in a mixture of DCM:MeOH (1:1, 6 mL) was stirred under rt overnight. The resulting solution was added with excess solid ammonium hexafluorophosphate (NH_4PF_6), and then stirred for another 0.5 h. 5 mL DCM was added into the solution, and the solution was washed by H_2O three times. The organic layer was filtered by 13 mm syringe filter to remove undissolved impurities. The filtrate was reduced in volume by rotary evaporation to generate solid. The solid was dissolved into acetone, and then the product was precipitated by adding diethyl ether, and filtered to yield desired product.

*Characteristics of complex **SM_1** and its ligands*

Complex **SM_1**: Yield: 33%. ^{1}H NMR (400 MHz, Acetone-d_6) δ 9.16 – 9.08 (m, 2H), 9.03 (dd, *J* = 8.6, 1.4 Hz, 2H), 8.51 (dt, *J* = 8.0, 0.9 Hz, 2H), 8.28 (dd, *J* = 4.9, 1.4 Hz, 2H), 8.06 – 7.96 (m, 4H), 7.97 – 7.88 (m, 4H), 7.48 (s, 3H), 7.25 (ddd, *J* = 8.1, 7.3, 1.3 Hz, 2H), 7.04 (td, *J* = 7.5, 1.3 Hz, 2H), 6.43 (dt, *J* = 7.7, 1.2 Hz, 2H), 2.92 (s, 6H). ^{13}C NMR (101 MHz, Acetone) δ 166.59, 150.14, 146.61, 145.47, 141.42, 138.07, 136.49, 133.88, 133.23, 132.71, 132.25, 131.20, 130.95, 130.15, 128.74, 127.39, 127.21, 127.09, 124.18, 122.53, 15.59. MALDI-TOF-HRMS: Calcd. for

$C_{44}H_{32}N_4Rh$ [M–PF_6]$^+$: 719.1682 Found: 719.1711. Anal.: ($C_{44}H_{32}N_4RhPF_6$) C, H, N: calcd. 61.12, 3.73, 6.48; found 61.40, 3.81, 6.52.

Complex **SM_2**: Reported[1]

Complex **C1**: Yield: 72%. ^{1}H NMR (400 MHz, Acetone-d_6) δ 9.07 (dd, J = 8.6, 1.4 Hz, 2H), 8.50 (dd, J = 4.9, 1.3 Hz, 2H), 8.31 (dt, J = 8.2, 1.1 Hz, 2H), 8.11 – 8.00 (m, 4H), 7.98 (d, J = 8.4 Hz, 2H), 7.62 (ddt, J = 5.7, 1.7, 0.9 Hz, 2H), 7.37 (dd, J = 8.3, 2.0 Hz, 2H), 7.10 (ddd, J = 7.3, 5.7, 1.4 Hz, 2H), 6.51 (dd, J = 2.0, 1.1 Hz, 2H), 2.92 (s, 6H). ^{13}C NMR (101 MHz, Acetone) δ 170.56, 164.86, 150.56, 150.35, 145.46, 144.06, 140.11, 136.82, 135.73, 133.37, 132.37, 127.73, 127.43, 127.35, 125.38, 125.11, 121.55, 15.57. MALDI-TOF-HRMS: Calcd. for $C_{36}H_{26}Br_2RhN_4$ [M–PF_6]$^+$: 776.9559 Found: 776.9598. Anal.: ($C_{36}H_{26}Br_2RhN_4PF_6$) C, H, N: calcd. 46.88, 3.10, 5.80; found 47.06, 3.10, 5.80.

Complex **Set7_1a**: Reported[2]

Fluorescence polarization assay

The fluorescence polarization assay is performed based on the proposal of SET7/9 SAM-Screener^{TM} Assay Kit. Shortly, a 384-well solid plate (low volume; black; Cayman, 400093) was used to perform to HEK293 cells were seeded in six well plates 24 h before transfection. 10 μL of SET7/9 (human recombinant) assay enzyme was incubated with 5 μL of positive control or negative control or complexes for 15 minutes at room temperature in a 384-well solid plate, respectively. Then, 5 μL of the reconstituted SAM-binding site probe was added to every well for 30 minutes at room temperature. The levels of were recorded using SpectraMax M5 microplate reader with excitation and emission wavelengths of 575 and 620 nm.

Knockdown assay

Cells were seeded in 6-well plate at 80% confluence in DMEM medium for 24h. Lipofectamine 3000 reagent and siRNA was gently mixed and incubated for 15 min at room temperature. 500 μL of siRNA-lipid complex were directly added to cells in 1.5 mL DMEM culture medium. Cells were then incubated at 37 °C in a CO_2 incubator for 72 h post-transfection before the further research.

Luciferase reporter assay

Cells were transiently transfected with pRL-TK and the ARE-luciferase reporter plasmids for 36 h. DHT-induced cells were treated with compounds (10 μM) for 8 h before measurement. Luciferase activity was measured using a spectrophotometer (Spectra-max M5, Molecular Devices, USA) and was integrated over a 10 second period. The results were standardized with the activity of Renilla luciferase.

Cellular thermal shift assay

Cellular thermal shift assay was performed to monitor the target engagement of **SM_1** in prostate cell lysates. Briefly, cell lysates from 2×10^6 cells were collected, diluted in PBS and separated in the same aliquots. Each aliquot was treated with **SM_1** (10 μM) or DMSO. 30 min after incubation at room temperature, the compound-treated lysates were divided into 50 μL in each of tubes and heated individually at different temperatures. The heated lysates were centrifuged and the supernatants were analyzed by SDS-PAGE followed by immunoblotting analysis by probing with the indicated antibody.

BiFC assay

Screening of inhibitors of the menin-MLL interaction was performed using the in vitro BiFC assay with MLL as our previous report.[3] Briefly, screening of inhibitors of the menin-MLL interaction was performed using the in vitro BiFC assay with MLL-VC210 and VN210-menin probes. MLL-VC210 and VN210-menin were co-transfected into HepG2 cells using TurboFect Transfection Reagent for 24 h. The cells were incubated with inhibitors at the indicated concentrations 16 h at 37 °C in 96-well microtiter plates. Nuclei were then stained with DAPI 1h. YFP signals and DAPI fluorescence were captured using IN Cell Analyzer 2000.

Immunoblotting assay

Cells were harvested to obtain whole-cell extracts by the addition of one volume of 250 mM Tris–HCl (pH 6.8), 20% glycerol, 2% sodium dodecyl sulfate (SDS), 5% 2-mercaptoethanol, and 0.2% bromophenol blue to cells in one volume of PBS followed by boiling for 5 min. Samples were resolved on sodium dodecyl sulfate polyacrylamide gel electrophoresis (SDS/PAGE) gels and transferred to polyvinylidene fluoride (PVDF) membranes. Blots were probed with antibodies to H3K4me3, p27, GAPDH and beta-actin (cell signaling). After incubation with secondary antibodies, blots were developed with ECL reagent (Thermo Fisher).

Co-immunoprecipitation (Co-IP) assay

The co-IP assay was performed as previously described.[4, 5] Briefly, cells were seeded at the density of 2×10^6 cells in a six-well plate. Cells were treated with the 10 μM **SM_1** for 8 h. Cells were lysed and collected as the protein samples. The concentration of protein samples was calculated using the Pierce BCA protein assay kit. 30 μg of each protein sample were incubated overnight with 10 μL pre-incubated anti-MLL and anti-SET7/9 magnetic beads according to the manufacturer's protocol. The complex was washed 5 times to elute non-specific and non-cross-

linked antibodies. Then, the precipitated proteins were subjected to SDS-PAGE and analyzed by Western blotting with the indicated antibodies.

Cell viability assay

The cytotoxicity of the compounds towards normal human cells were measured by an MTT assay. Cells were seeded at a density of 5,000 cells per well in 96-well plates and incubated for 12 h under hypoxia. Compounds dissolved in DMSO were added to cells followed by incubation for 48 h. Then 10 μL of 5 mg/mL MTT (3-(4,5-dimethylthiazol-2-yl)-2,5-diphenyltetrazolium bromide) reagent was added to each well. After 4 h incubation in the dark, 100 μL of DMSO was added to each well, and the intensity of absorbance was then determined by a SpectraMax M5 microplate reader at a wavelength of 570 nm.

Cell cycle arrest assay

DHT-induced cells (1×10^6 cells/well) were incubated with indicated concentrations of complex **SM_1**, MDV3100 or DMSO for 16 h. Cells were collected and fixed in 1.0 ml aqueous ethanol (70%, v/v) overnight, and then stained by PI (50 μg/mL) and RNase A (50 μg/mL) for 30 min. Cell cycle arrest was then analyzed by using the BD Accuri™ C6 Plus flow cytometer.

Drug studies in tumor xenograft model

Male nude mice (6-8 weeks) were purchased from The Chinese University of Hong Kong. The "Institutional Animal Care and User Committee guidelines" of Macau University of Science and Technology were followed for all animal experiments. Male nude mice were randomly divided into five groups and subcutaneously injected with LNCaP cells (2×10^6 cells) resuspended in 50 μl of serum-free medium plus 50 μl of Matrigel (Corning, New York, USA). Complex **SM_1** (0.5

mg/kg, 1 mg/kg, 2 mg/kg) and Taxol (10 mg/kg) were dissolved in polyethylene glycol 400: ethanol: ddH_2O= 6:1:3, and given by i.p. injection for 14 days. Body weight and tumor volumes (length × width^2 × 1/2) were measured every day.

Terminal dUTP nick-end labeling staining

Apoptosis in mouse testes was examined using TUNEL assay following the protocols of the apoptosis detection kit (C1086, Beyotime). The imaging was carried out by a Leica TCS SP8 confocal laser scanning microscope system.

Statistical analysis

Data were analyzed using GraphPad Prism software with one-way analysis of variance (ANOVA) unless otherwise noted. Data were presented as mean ± SD (standard deviation) unless otherwise noted. $P < 0.05$ was considered statistically significant.

Supplementary Figures

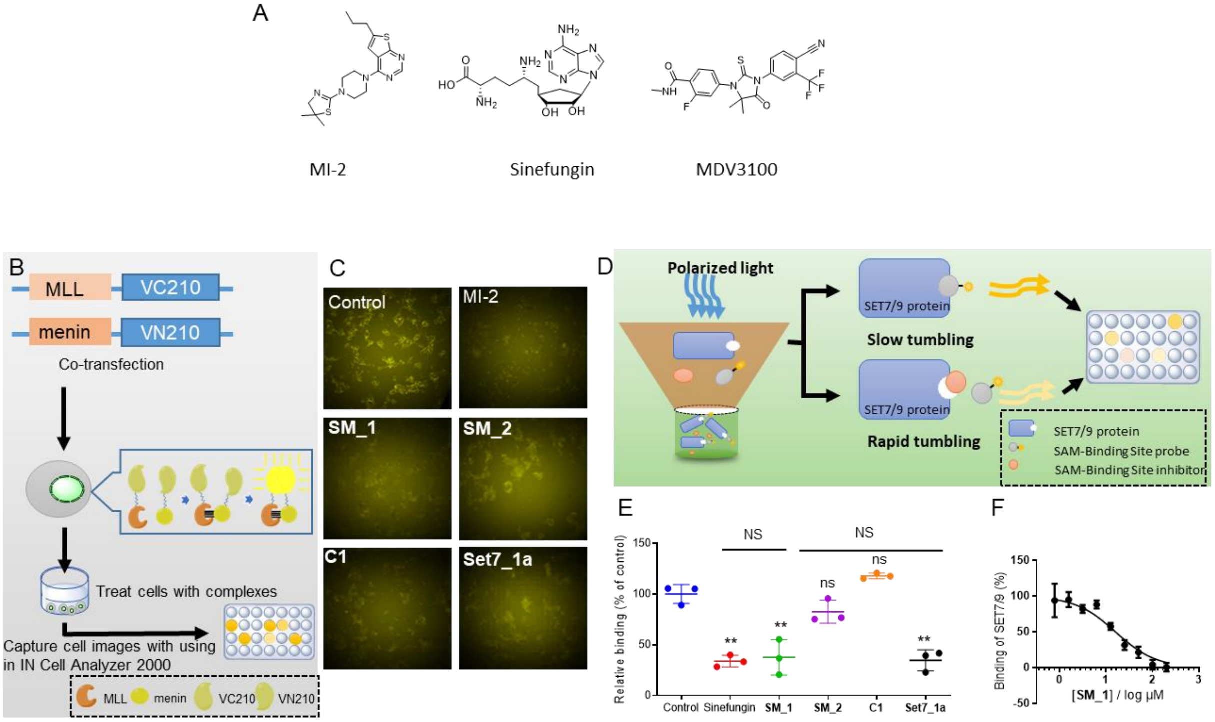

Figure S1. Screening of small molecules as potential SET7 and MLL complex dual inhibitors. (A) Chemical structures of positive controls MI-2, sinefungin and MDV3100. (B) Schematic diagram of the BiFC assay using MLL-VC210 and VN210-menin co-transfected HEK293 cells. (C) BiFC images comparing activities of complexes **SM_1**, **SM_2**, **Set7_1a**, **C1**, and MI-2 (10 μM) for disruption of the menin–MBM1 interaction. (D) Schematic diagram of the FP assay. (E) Effect of complexes **SM_1**, **SM_2**, **Set7_1a**, **C1**, and sinefungin on the interaction of between SAM and its binding pocket of SET7. (F) Complex **SM_1** inhibits SET7 activity. $^{ns}P > 0.05$, $^{**}P < 0.01$ vs. Control, $^{NS}P > 0.05$ vs. **SM_1** group.

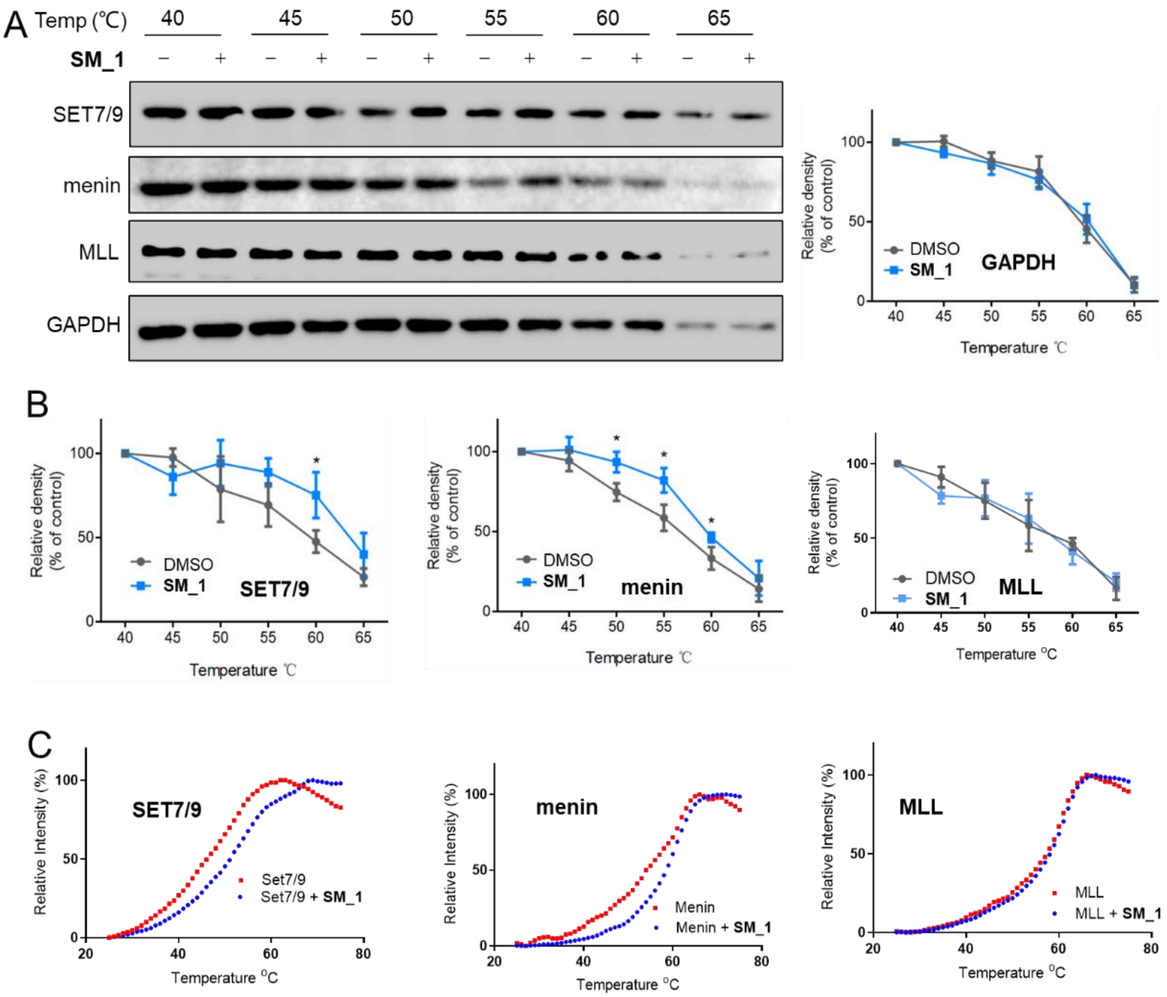

Figure S2. Complex **SM_1** selectively engages SET7 and memin *in cellulo*. (A) Stabilization of SET7, menin, MLL and GAPDH in CRPC cell lysates by **SM_1**. (B) Densitometry analysis of the proteins by using cell-based Western blot. (c) FTS analysis of the potential target proteins in presence of complex **SM_1** (10 μM). **P* < 0.05, ***P* < 0.01 vs. DMSO.

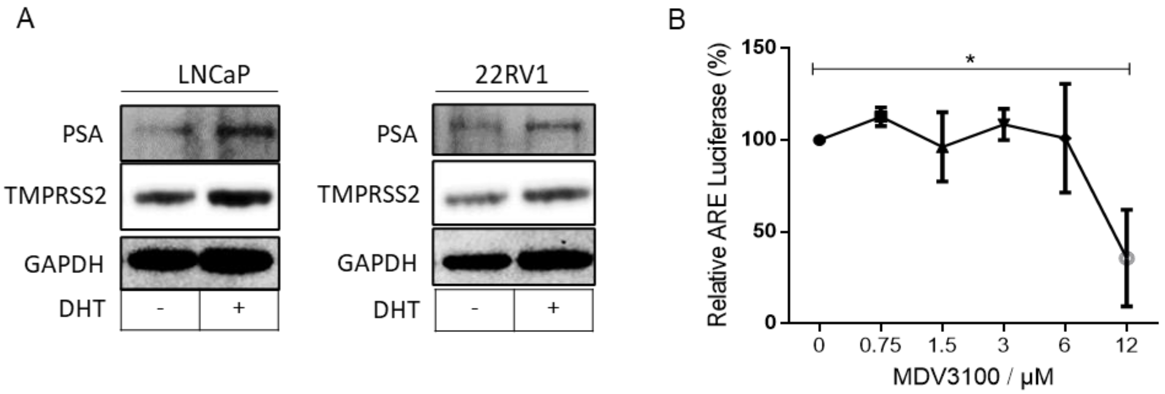

Figure S3. Complex **SM_1** inhibits ARE-related transcription activity by disrupting the interactions between MLL and SET7 with AR. (A) The effect of DHT on the transcriptional activity of the AR in LNCaP and 22RV1 cell lines. (B) Dose–response effect of MDV3100, a reported AR inhibitor, on ARE activity as measured by the luciferase reporter assay. IC_{50} value: *ca.* 10.0 μM as measured by the luciferase reporter assay. P values were calculated using a two-sided t-test. *$P < 0.05$ vs. DMSO group.

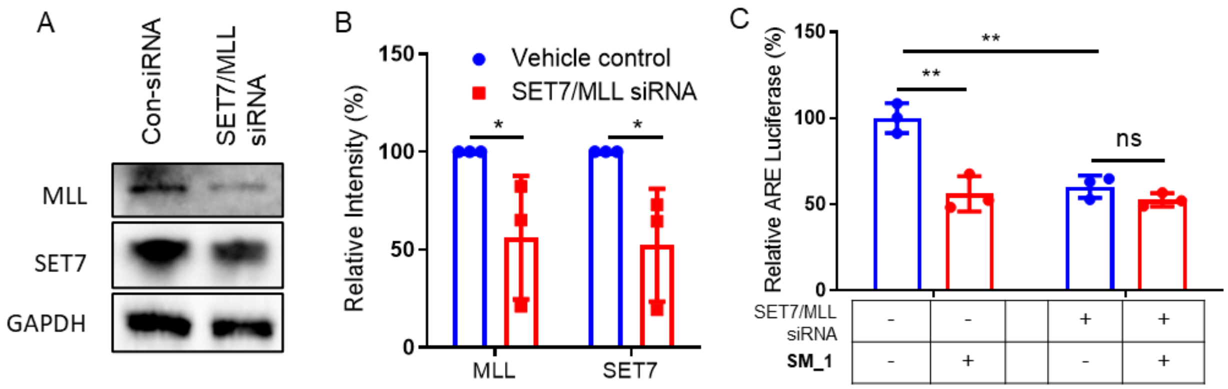

Figure S4. Complex **SM_1** inhibits the transcriptional activity of the AR by selectively targeting MLL and SET7 in DHT-induce LNCaP cells. (A) SET7 and MLL are down-regulated after siRNA pre-treatment. (B) Densitometry analysis of SET7 and MLL levels on the Western blot after siRNA pretreatment. (C) ARE transcription activity in LNCaP cells with or without knockdown SET7/MLL in the presence or absence of 3 μM of complex **SM_1**. *$P < 0.05$, **$P < 0.01$. ns means not significant.

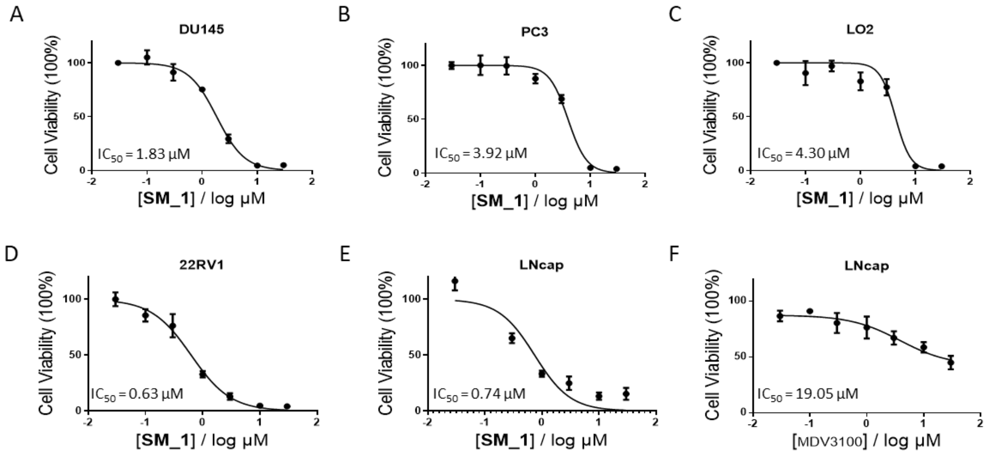

Figure S5. The cytotoxicity effect of **SM_1** on prostate cancer cells and normal cells. (A) DU145 cells, (B) PC3 cells, (C) LO2 cells, (D) 22RV1cells, and (E) LNCaP cells. (F) The cytotoxicity effect of MDV3100 on LNCaP cells. Cells were exposed to the indicated concentrations of **SM_1** or MDV3100 for 72 h. Each sample was run in triplicate.

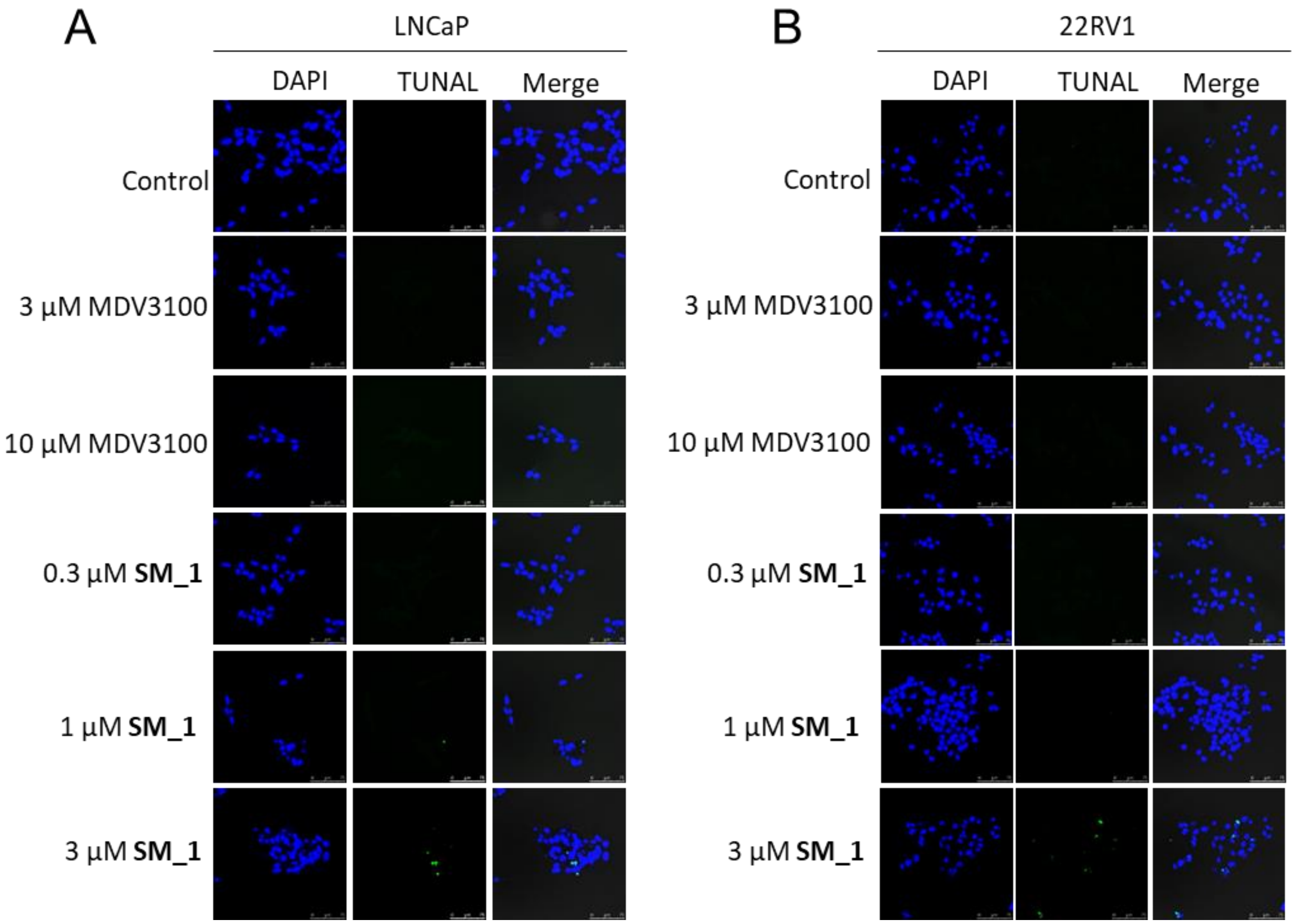

Figure S6. Effects of complex **SM_1** or MDV3100 on cell apoptosis in CRPC cell lines LNCaP (A) and 22RV1 (B).

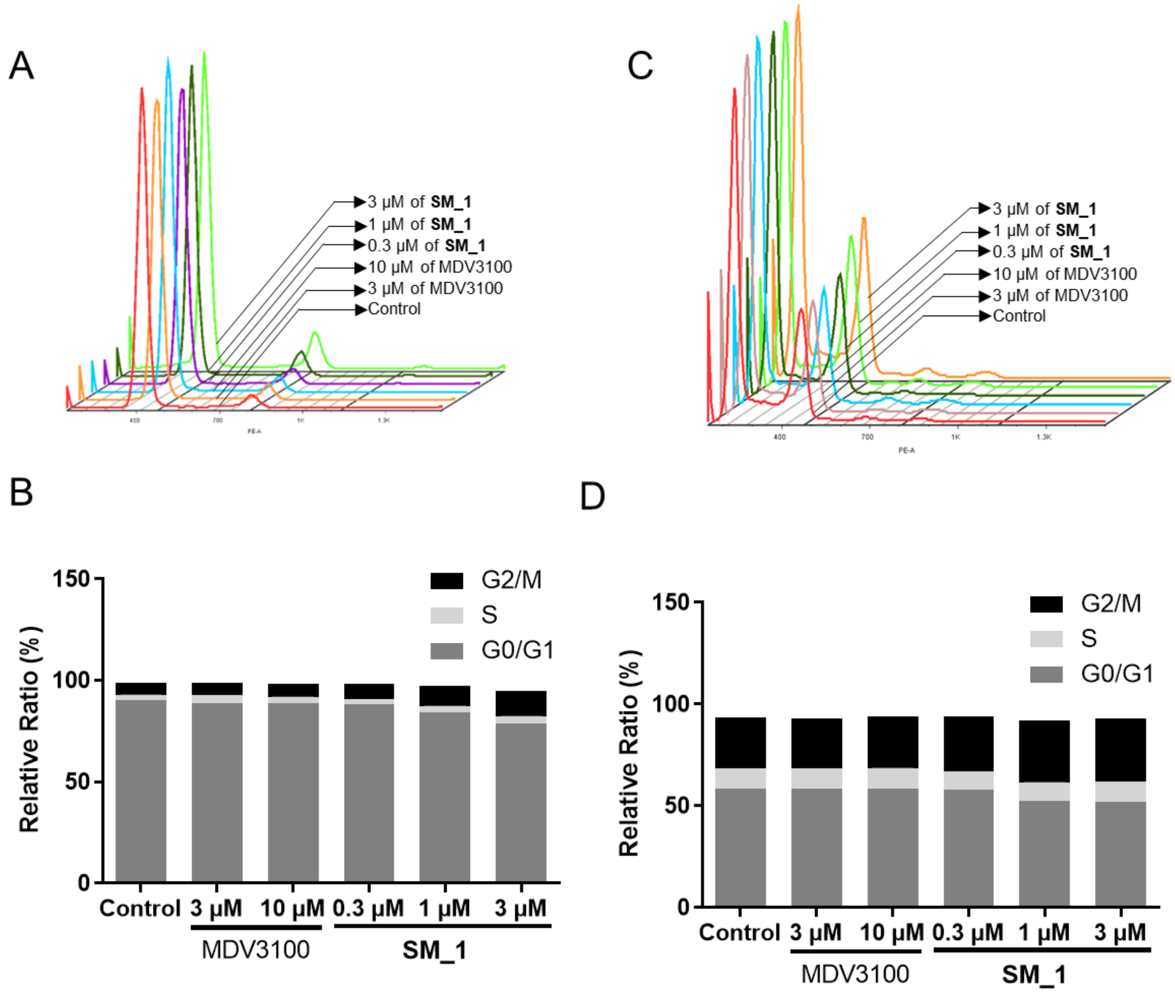

Figure S7. A dual SET7/MLL small molecule inhibitor impairs CRPC tumor growth *in cellulo* and *in vivo*. Effects of complex **SM_1** or MDV3100 on cell cycle were examined with PI staining in human CPRC cell line LNCaP (A and B) and 22RV1 (C and D).

References

1 Lu L, Chan DS-H, Kwong DWJ, He H-Z, Leung C-H, Ma D-L. Detection of nicking endonuclease activity using a G-quadruplex-selective luminescent switch-on probe. Chemical Science 2014; 5: 4561-8.

2 Li G, Li D, Wu C, Li S, Chen F, Li P, *et al*. Homocysteine-targeting compounds as a new treatment strategy for diabetic wounds via inhibition of the histone methyltransferase SET7/9. Experimental & Molecular Medicine 2022; 54: 988-98.

3 Zhong H-J, Lee BR, Boyle JW, Wang W, Ma D-L, Chan PWH, *et al*. Structure-based screening and optimization of cytisine derivatives as inhibitors of the menin–MLL interaction. Chemical Communications 2016; 52: 5788-91.

4 Bol GM, Vesuna F, Xie M, Zeng J, Aziz K, Gandhi N, *et al*. Targeting DDX3 with a small molecule inhibitor for lung cancer therapy. EMBO Mol. Med. 2015; 7: 648-69.

5 Yang C, Wang W, Chen L, Liang J, Lin S, Lee MY, *et al*. Discovery of a VHL and HIF1alpha interaction inhibitor with in vivo angiogenic activity via structure-based virtual screening. Chem. Commun. (Camb.) 2016; 52: 12837-40.
